# Identification of Markers that Distinguish Monocyte-Derived Fibrocytes from Monocytes, Macrophages, and Fibroblasts

**DOI:** 10.1371/journal.pone.0007475

**Published:** 2009-10-16

**Authors:** Darrell Pilling, Ted Fan, Donna Huang, Bhavika Kaul, Richard H. Gomer

**Affiliations:** Department of Biochemistry and Cell Biology, Rice University, Houston, Texas, United States of America; BMSI-A*STAR, Singapore

## Abstract

**Background:**

The processes that drive fibrotic diseases are complex and include an influx of peripheral blood monocytes that can differentiate into fibroblast-like cells called fibrocytes. Monocytes can also differentiate into other cell types, such as tissue macrophages. The ability to discriminate between monocytes, macrophages, fibrocytes, and fibroblasts in fibrotic lesions could be beneficial in identifying therapies that target either stromal fibroblasts or fibrocytes.

**Methodology/Principal Findings:**

We have identified markers that discriminate between human peripheral blood monocytes, tissue macrophages, fibrocytes, and fibroblasts. Amongst these four cell types, only peripheral blood monocytes express the combination of CD45RO, CD93, and S100A8/A9; only macrophages express the combination of CD45RO, 25F9, S100A8/A9, and PM-2K; only fibrocytes express the combination of CD45RO, 25F9, and S100A8/A9, but not PM-2K; and only fibroblasts express the combination of CD90, cellular fibronectin, hyaluronan, and TE-7. These markers are effective both *in vitro* and in sections from human lung. We found that markers such as CD34, CD68, and collagen do not effectively discriminate between the four cell types. In addition, IL-4, IL-12, IL-13, IFN-γ, and SAP differentially regulate the expression of CD32, CD163, CD172a, and CD206 on both macrophages and fibrocytes. Finally, CD49c (α3 integrin) expression identifies a subset of fibrocytes, and this subset increases with time in culture.

**Conclusions/Significance:**

These results suggest that discrimination of monocytes, macrophages, fibrocytes, and fibroblasts in fibrotic lesions is possible, and this may allow for an assessment of fibrocytes in fibrotic diseases.

## Introduction

There are multiple sources of fibroblast-like cells present in fibrotic lesions and healing wounds [1]–[5]. In addition to the proliferation of resident fibroblasts, bone marrow-derived hematopoietic precursors present within the blood are attracted to sites of injury where they differentiate into spindle-shaped fibroblast-like cells called fibrocytes, and at least in part, mediate tissue repair and fibrosis [6]–[14].

Fibrocytes appear to differentiate from CD14+ peripheral blood monocytes [7], [15]–[18]. Mature fibrocytes express markers of both hematopoietic cells (CD34, CD43, CD45, LSP-1, MHC class II) and stromal cells (collagen I and III) [6]–[9], [16], [19]. Fibrocytes also express the chemokine receptors CCR2, CCR7, and CXCR4, which regulate their entry into inflammatory lesions [7], [16], [20]–[23]. Mature fibrocytes exposed to TGF-β *in vitro* are able to develop further into myofibrocytes, a myofibroblast-like population of cells that express α-SMA and are able to contract collagen gels, in an *in vitro* model of wound contraction [7].

At present, there is no single specific marker for fibrocytes. The combination of intracellular collagen staining and the expression of CD45 or LSP-1, plus either CD34 or CXCR4, has been considered as a sufficiently accurate criterion to discriminate fibrocytes from leukocytes, dendritic cells, endothelial cells and tissue-resident fibroblasts *in vitro* and *in vivo* [8], [9]. However, several groups have shown that the progressive loss of CD34 and eventually CD45 on fibrocytes can lead to an underestimation of fibrocyte numbers [8], [9], [24]. These data suggest that some combinations of markers to identify fibrocytes do not adequately discriminate different cell populations, and may lead to either over or underestimation of fibrocyte numbers [24]. Another problem with the use of CD34, CD45, CXCR4, LSP-1, and collagen is that even combinations of these antibodies define overlapping cell types. CD45 and LSP-1 are expressed by all hematopoietic cells including lymphocytes, monocytes, macrophages, and fibrocytes [16], [25], [26]. CXCR4 is expressed by T cells, macrophages, inflammatory fibroblasts, as well as fibrocytes [27]–[30]. Besides fibrocytes, CD34 is expressed by hematopoietic precursors, endothelial cells, stromal cells, and may be expressed by macrophages and dendritic cells [6], [31]–[33]. The detection of fibrocytes using intracellular collagen is further complicated, as fibrocytes only produce low levels of collagen, and under certain circumstances macrophages may also produce collagen, as well as other extracellular matrix proteins [10], [34]–[38]. Macrophages may also ingest collagen, leading to a false positive identification of collagen production [39]. These data indicate that other markers are required to separate fibrocytes from macrophages.

Another major difficulty in analyzing the role of fibrocytes in fibrotic diseases is the inability to identify markers specific for fibroblasts [40]–[47]. Fibroblast subsets can be identified from within the same tissue or from distinct anatomical sites due to the expression of different adhesion molecules, extracellular matrix, chemokine, cytokine and HOX proteins [41]–[43], [48]. Fibroblasts also differentially express molecules, including CD248/endosialin, ER-TR7, and gp38/podoplanin, during development and inflammation [42], [46], [49]. Compounding these issues, markers that initially appeared to be fibroblast-specific such as FSP-1/S100A4 have since been reported to be more widely expressed [47], [50]. Finally, markers originally defined as specific for monocytes and macrophages such as CD68 and Mac-2/galectin-3, also label fibroblasts [51]–[54].

In this report, we have screened freshly isolated human peripheral blood monocytes (10–15 µm cells that express CD14 with a bilobed nucleus), *in vitro*-cultured macrophages (15–20 µm cells, with a large nucleus and pronounced cytoplasm, derived from peripheral blood monocytes), *in vitro* cultured fibrocytes (50–200 µm long spindle-shaped cells with an oval nucleus derived from peripheral blood monocytes, that express a variety of hematopoietic markers such as CD34, CD45, LSP-1, as well as collagen), and *in vitro* cultured fibroblasts (stellate-shaped cells derived from connective tissue, that produce many extracellular matrix proteins and do not express hematopoietic markers) with commercially available reagents to identify markers that can accurately discriminate between these populations.

## Methods

### Cell culture conditions and fibrocyte differentiation assay

Human peripheral blood was collected into heparin vacutainer tubes (#367874; BD Bioscience, Franklin Lakes, NJ) from healthy adult volunteers with specific approval of Rice University's Institutional Review Board. Written consent was received and all samples were de-identified before analysis. Peripheral blood mononuclear cells (PBMC) were isolated by Ficoll-Paque Plus (GE Healthcare Biosciences, Piscataway, NJ) as described previously [16]–[18]. PBMC were cultured in serum-free medium (SFM), which consists of FibroLife basal media (LM-0001, Lifeline Cell Technology, Walkersville, MD), supplemented with 10 mM HEPES (Sigma-Aldrich, St. Louis, MO), 1x non-essential amino acids (Sigma-Aldrich), 1 mM sodium pyruvate (Sigma-Aldrich), 2 mM glutamine (Invitrogen, Carlsbad, CA), 100U/ml penicillin, 100 µg/ml streptomycin (Sigma-Aldrich), and 1x ITS-3 (Sigma-Aldrich). PBMC were cultured in flat-bottomed 96 well tissue culture plates or eight well glass microscope slides (177402, Lab-Tek, Nalge Nunc International, Naperville, IL) in 200 µl or 400 µl per well respectively, at 2.5×10^5^ cells per ml in a humidified incubator containing 5% CO_2_ at 37°C. Immediately following Ficoll separation PBMC incubated in 8-well glass slides for 1 hour at 37°C were used to analyze, by immunohistochemistry, the expression of markers on monocytes *ex vivo*. PBMC were also cultured for 7 days in the presence of 10% fetal calf serum (FCS; PAA Laboratories, New Bedford, MA)and 10 ng/ml M-CSF (Peprotech, Rocky Hill, NJ), which are standard conditions for the culture of human macrophages [55]–[58]. Cytokines (all from Peprotech) were added at 10 ng/ml. SAP (EMD-Calbiochem, San Diego, CA) was added at 1 µg/ml. Fibrocytes were defined as adherent spindle-shaped cells with an oval nucleus, as described previously [16]–[18].

Normal human dermal fibroblasts (Lonza, Walkersville, MD) and MRC-5 fetal fibroblasts (ATCC, Manassas, VA) were cultured in RPMI-1640 (Sigma-Aldrich) supplemented with 10% FCS, 100U/ml penicillin, 100 µg/ml streptomycin, and 2 mM glutamine, as described previously [59], [60]. Human umbilical vein endothelial cells (HUVEC, Lonza) were cultured in EGM-2 medium (Lonza).

### Histology and immunohistochemistry

Serial 5 µm thick sections from patients with Chronic Obstructive Pulmonary Disease (COPD) or Interstitial Lung Disease (ILD) were obtained from the National Heart Lung and Blood Institute-sponsored Lung Tissue Research Consortium (LTRC), with specific approval of Rice University's Institutional Review Board. Written consent was received and all samples were de-identified before analysis. Sections were either standard formalin fixed paraffin-embedded sections or “HOPE fixed” before paraffin-embedding [61]. Formalin fixed slides were dewaxed with xylene, then rehydrated through a graded series of alcohols and distilled water. HOPE-fixed slides were treated with 60°C isopropanol for 10 minutes, then treated with fresh 60°C isopropanol for a further 15 minutes. Slides were rehydrated in 70% acetone (v/v in distilled water), and then distilled water. Human PBMC cultured on eight well glass microscope slides were air dried for at least 60 minutes before fixation in acetone for 15 minutes at room temperature.

For all slides, non-specific binding was blocked by incubation in PBS containing 4% BSA (PBS-BSA) for 60 minutes. Endogenous biotin was blocked by the addition of streptavidin and biotin solutions in PBS-BSA, following the manufacturers' instructions (Streptavidin/Biotin Blocking Kit, Vector Laboratories, Burlingame, CA). Slides were then incubated with 5 µg/ml primary antibodies in PBS-BSA for 60 minutes (see Table 1 for antibody list), as described previously [13], [16], [18], [60]. Isotype-matched irrelevant mouse and rat monoclonal antibodies (BD-Biosciences), irrelevant rabbit polyclonal antibodies (Jackson ImmunoResearch, West Grove, PA) or goat polyclonal antibodies (R&D Systems, Minneapolis, MN), at 5 µg/ml in PBS-BSA were used as controls. Primary antibodies were detected with either biotinylated goat F(ab')_2_ anti-mouse IgG, biotinylated goat F(ab')_2_ anti-rabbit IgG (both cross-adsorbed against human Ig, Southern Biotechnology, Birmingham, AL), biotinylated mouse F(ab')_2_ anti-rat IgG, or donkey F(ab')_2_ anti-goat IgG (both cross-adsorbed against human and murine serum proteins, Jackson ImmunoResearch). All secondary antibodies were used at 2.5 µg/ml in PBS-BSA for 30 minutes. Biotinylated antibodies were detected by a 1/200 dilution of ExtrAvidin alkaline phosphatase (Sigma-Aldrich or Vector Laboratories) in PBS-BSA. Staining was developed with the Vector Red Alkaline Phosphatase Kit (Vector Laboratories) for 7 minutes. Sections were then counterstained for 10 seconds with Gill's hematoxylin #3 (Sigma-Aldrich) diluted 1∶5 with water, rinsed in water, and then treated with Scott's tap water substitute (20 g/L MgSO_4_^.^7H_2_O; 2 g/L NaHCO_3_) for 30 seconds. Slides were then dehydrated through 70%, 95%, and 100% ethanol, cleared with xylene, and mounted with VectaMount (Vector Laboratories). All procedures were performed at room temperature. Non-specific esterase (α-Naphthyl Acetate) staining was performed according to the manufacturer's protocol (α-Naphthyl Acetate kit, Sigma-Aldrich).

**Table 1:** List of antibodies used for staining.

Marker	Clone or Catalog number	Isotype	Source
CD9	M-L13	Mouse IgG1	BD-Biosciences
CD10	SN5c	Mouse IgG1	AbD Serotec, Raleigh, NC
CD11a	HI111	Mouse IgG1	BD-Biosciences
CD11b	ICRF44	Mouse IgG1	BD-Biosciences
CD11c	3.9	Mouse IgG1	BioLegend
CD13	WM15	Mouse IgG1	BD-Biosciences
CD14	HDC14	Mouse IgG1	BioLegend
CD16	GRM1	Mouse IgG2a	Southern Biotechnology
CD18	6.7	Mouse IgG1	BD-Biosciences
CD19	HIB19	Mouse IgG1	eBioscience, San Diego, CA
CD21	FR5A10	Mouse IgG1	Lab Vision, Fremont, CA
CD29	TDM29	Mouse IgG1	Southern Biotechnology
CD31	WM59	Mouse IgG1	BioLegend
CD32	FLI8.26	Mouse IgG2b	BD-Biosciences
CD32	AT10	Mouse IgG1	AbD Serotec
CD32a	AF1875	Goat polyclonal	R&D Systems
CD32b	AF1330	Goat polyclonal	R&D Systems
CD33	WM53	Mouse IgG1	AbD Serotec
CD34	QBend10	Mouse IgG1	BeckmanCoulter, Miami, FL
CD35	E11	Mouse IgG1	Lab Vision
CD36	SM0	Mouse IgM	AbD Serotec
CD41	HIP8	Mouse IgG1	BioLegend
CD43	1G10	Mouse IgG1	BD-Biosciences
CD44	515	Mouse igG1	BD-Biosciences
CD45	HI30	Mouse IgG1	BD-Biosciences
CD45RA	HI100	Mouse IgG1	BioLegend
CD45RB	MEM-55	Mouse IgG2b	BioLegend
CD45RO	UCHL1	Mouse IgG2a	BD-Biosciences
CD45-B220	RA3-6B2	Rat IgG2a	BD-Biosciences
CD49a	SR84	Mouse IgG1	BD-Biosciences
CD49b	AK7	Mouse IgG1	Millipore
CD49c	C3 II.1	Mouse IgG1	BD-Biosciences
CD49d	HP2/1	Mouse Igg1	Millipore
CD49e	SAM-1	Mouse IgG2b	Millipore
CD49f	GoH3	Rat IgG2a	BD-Biosciences
CD51/61	VI-PL2	Mouse IgG1	BD-Biosciences
CD64	10.1	Mouse IgG1	BD-Biosciences
CD68	Y1/82A	Mouse IgG2b	BD-Biosciences or BioLegend
CD81	JS-81	Mouse IgG1	BD-Biosciences
CD90 (Thy1)	5E10	Mouse IgG1	BD-Biosciences
CD91	A2MR-α2	Mouse IgG1	BD-Biosciences
CD93	AF2379	Goat polyclonal	R&D Systems
CD94	HP-3D9	Mouse IgG1	BD-Biosciences
CD104	450-9D	Mouse IgG1	BD-Biosciences
CD105	SN6	Mouse IgG1	AbD Serotec
CD106	STA	Mouse IgG1	BioLegend
CD115	3-4A4	Rat IgG2b	Santa Cruz Biotechnology, CA
CD117	YB5.B8	Mouse IgG1	BD-Biosciences
CD133	293C3	Mouse IgG2b	Miltenyi Biotec Inc., Auburn, CA
CD141	1A4	Mouse IgG1	BD-Biosciences
CD150	A12 (7D4)	Mouse IgG1	BioLegend
CD163	GHI/61	Mouse IgG1	BD-Biosciences
CD164	67D2	Mouse IgG1	BioLegend
CD166	3A6	Mouse IgG1	BD-Biosciences
CD169	HSn 7D2	Mouse IgG1	Santa Cruz Biotechnology
CD172a	SE5A5	Mouse IgG1	BioLegend
CD172b	B4B6	Mouse IgG1	BD-Biosciences
CD180	MHR73-11	Mouse IgG1	BioLegend
CD206	15-2	Mouse IgG1	BioLegend
CD209	DCN46	Mouse IgG2b	BD-Biosciences
CD248 (Endosialin)	N-16	Goat polyclonal	Santa Cruz Biotechnology
CD280	E-17	Goat polyclonal	Santa Cruz Biotechnology
CD328 (Siglec-7)	F023-420	Mouse IgG1	BD-Biosciences
CD329 (Siglec-9)	E10-286	Mouse IgG1	BD-Biosciences
CCR2	48607	Mouse IgG2b	R&D Systems
CCR7	150503	Mouse IgG2a	R&D Systems
CXCR4	44716	Mouse IgG2b	R&D Systems
CX3CR1	TP502	Polyclonal rabbit IgG	Torrey Pines BioLabs
Cellular Fibronectin	Fn-3	Mouse IgG1	Cymbus Biotechnology
Collagen-I	COL-1	Mouse IgG1	Sigma-Aldrich
Collagen-I	600-401-103	Polyclonal rabbit IgG	Rockland Inc
Collagen-III	600-401-105	Polyclonal rabbit IgG	Rockland Inc
Collagen-IV	600-401-106	Polyclonal rabbit IgG	Rockland Inc
Pro-Collagen I	M-58	Rat IgG1	Millipore, Temecula, CA:
Cytokeratin	C-11	Mouse IgG1	Sigma-Aldrich
Desmin	DE-U-10	Mouse IgG1	Sigma-Aldrich
Fibroblasts-Reticular	ER-TR7	Rat IgG2a	Cedarlane, Burlington, NC
Fibroblasts-Thymic	TE-7	Mouse IgG1	Millipore
FAP	F11-24	Mouse IgG1	Bender Medsystems, Burlingame, CA
Fibronectin	F3648	Polyclonal rabbit IgG	Sigma-Aldrich
Bio HA-BP	400763-1A	Biotinylated protein	Associates of Cape Cod, MA
Lamin B	C-20	Goat polyclonal	Santa Cruz Biotechnology
LSP-1	16	Mouse IgG1	BD-Biosciences
LYVE	AF2089	Goat polyclonal	R&D Systems
Mac-2/Galectin-3	eBioM3/38	Rat IgG2a	eBioscience
Macrophage-PM-2K	PM-2K	Mouse IgG1	AbD Serotec
Macrophage- S100A8/A9	Mac387	Mouse IgG1	AbD Serotec
Macrophage-25F9	25F9	Mouse IgG1	eBioscience
Myeloid Ag	BM-1	Mouse IgG1	BioLegend
Prolyl-4-hydroxylase	3-2B12	Mouse IgG1	Millipore
PU.1	Sc-352	Polyclonal rabbit IgG	Santa Cruz Biotechnology
α-SMA	1A4	Mouse IgG2a	Sigma-Aldrich
STRO-1	STRO-1	Mouse IgM	DSHB/NIH, University of Iowa
Tenascin	BC-24	Mouse IgG1	Sigma-Aldrich
Tubulin	YL1/2	Rat IgG2a	Novus Biologicals, Littleton, CO
Vimentin-mesenchymal	LN-6	Mouse IgM	Sigma-Aldrich
Vimentin – all cells	VIM-13.2	Mouse IgM	Sigma-Aldrich
vWF		Goat polyclonal	Bethyl Labs, Montgomery, TX
Peanut agglutinin (PNA)	B-1075	Biotinylated lectin	Vector Laboratories
Soybean agglutinin (SBA)	B-1015	Biotinylated lectin	Vector Laboratories
Wheatgerm agglutinin (WGA)	B-1025	Biotinylated lectin	Vector Laboratories

For immunofluorescence staining, following incubation with primary antibodies as described above, slides were labeled with biotinylated goat F(ab')_2_ anti-mouse IgG (Southern Biotechnology), Rhodamine Red-X donkey F(ab')_2_ anti-rabbit IgG (Jackson ImmunoResearch), or NL637 donkey anti-goat IgG (R&D Systems). After washing, the biotinylated antibodies were detected with Streptavidin-Alexa 488 (Invitrogen) in PBS-BSA. Sections were then fixed for 30 minutes in 70% ethanol containing 0.1% Sudan Black B (Fisher Scientific, Pittsburgh, PA) to quench tissue autofluorescence [13]. Slides were then washed in PBS and mounted with VectaShield containing DAPI (Vector Laboratories).

For staining using both mouse IgG1 and IgG2a monoclonal antibodies, slides were first incubated with mouse IgG2a antibodies (UCHL1, CD45RO) and rabbit anti-collagen antibodies. After washing, the slides were incubated with Alexa-488 goat anti-mouse IgG (Invitrogen) and Rhodamine Red-X donkey F(ab')_2_ anti-rabbit IgG. After washing, the slides were incubated for at least 60 minutes with PBS containing 50% normal mouse serum (Gemini BioProducts, Woodland, CA) and 2% BSA. After washing, the slides were then incubated with mouse IgG1 monoclonal antibodies, and then incubated with isotype-specific biotinylated goat F(ab')_2_ anti-mouse IgG1 (Southern Biotechnology). After washing, the biotinylated antibodies were detected with Streptavidin-Alexa 647 (Invitrogen). Sections were then fixed for 30 minutes in 70% ethanol containing 0.1% Sudan Black B, washed in PBS, and mounted with VectaShield containing DAPI. All procedures were at room temperature.

### Flow cytometry

PBMC were analyzed by flow cytometry as described previously [18], [26], [59], [60], [62], [63]. Briefly, human PBMC were incubated with 5 µg/ml primary antibodies diluted in PBS-BSA for 30 minutes on ice. Isotype-matched irrelevant mouse monoclonal antibodies (BD-Biosciences) at 5 µg/ml in PBS-BSA were used as controls. PBMC were then washed twice in ice-cold PBS, before staining on ice for 30 minutes with 2 µg/ml FITC-conjugated goat F(ab')_2_ anti-mouse IgG (cross-adsorbed against human Ig, Southern Biotechnology) in PBS-BSA. For 3 color flow cytometry, PBMC were then incubated in PBS-BSA containing 20% normal mouse serum (Gemini BioProducts, West Sacramento, CA) for 30 minutes on ice, to block any remaining anti-mouse IgG binding sites. PBMC were then washed twice in ice-cold PBS and then stained on ice for 30 minutes with CD16-PE (BD-Biosciences) and CD14-PE/Cy7 (BioLegend, San Diego, CA) in PBS-BSA containing 20% normal mouse serum. PBMC were analyzed by flow cytometry using an Accuri C6 cytometer (Accuri, Ann Arbor, MI). Peripheral blood monocytes were identified by their size, granularity, and expression of CD14. At least 5,000 CD14 positive monocytes were examined for each marker.

### Image Analysis

Immunohistochemistry images were captured on an Axioskop microscope (Zeiss, Thornwood, NY) using an AxioCam MRc5 digital camera (Zeiss) and AxioVision software (Zeiss). Immunofluorescence images were captured on an Axioplan2 microscope (Zeiss) with a CoolSNAP HQ digital camera (Photometrics, Tucson, AZ) and Metamorph software (Molecular Devices, Downingtown, PA). Images were analyzed with ImageJ (Rasband, WS, NIH, Bethesda, MD).

### Statistics

Statistical analysis was performed using GraphPad Prism 4 software (GraphPad, San Diego, CA). Differences between multiple groups were assessed by ANOVA using Tukey's post-test. Significance was defined as p<0.05. In the figures, *** indicates p<0.001.

## Results

### Expression of markers on fibrocytes cultured for one week

**Figure 1:**
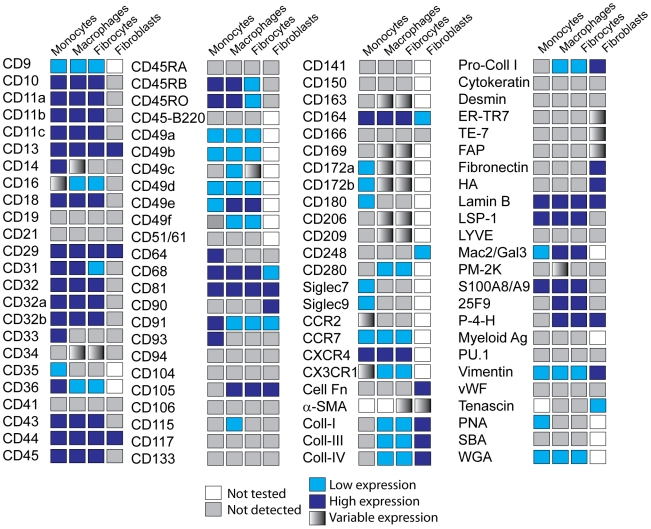
Expression of markers on monocytes, macrophages, fibrocytes, and fibroblasts. Combined data on the expression of markers on the cells analyzed.

**Figure 2:**
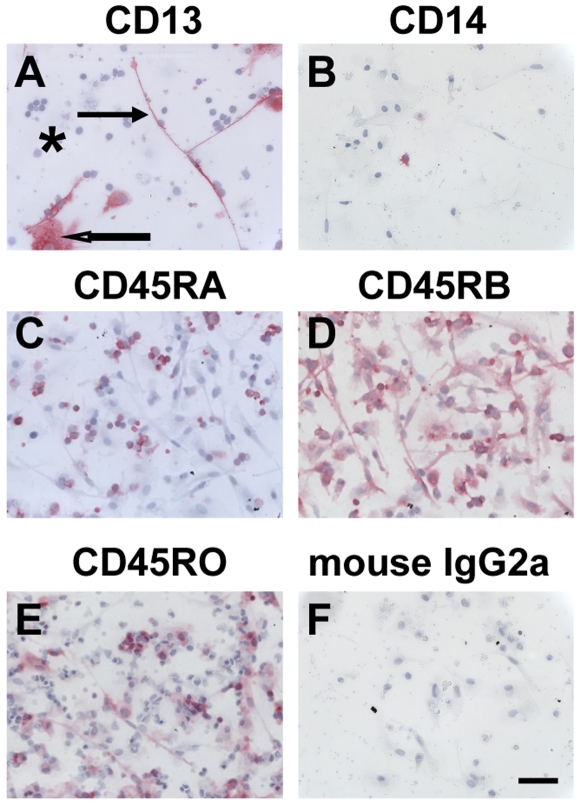
Expression of CD13, CD14, and CD45 isoforms on fibrocytes. PBMC were cultured in SFM at 5×10^5^ cells/ml in 8-well glass slides for 7 days. Cells were then air-dried, fixed, and stained with antibodies. A) CD13, B) CD14, C) CD45RA, D) CD45RB, E) CD45RO, F) Irrelevant mouse IgG2a control. Cells were then counterstained with hematoxylin to identify nuclei. Positive staining was identified by red staining, with nuclei counterstained blue. Solid arrow points to a fibrocyte, open arrow points to a macrophage, and asterisk indicates a cluster of lymphocytes. Photomicrographs are representative results from at least four different donors. Bar is 50 µm.

Fibrocytes, either cultured under a variety of conditions *in vitro*, or isolated from tissues *in vivo*, have been identified with a variety of markers, including LSP-1, α-SMA, CD13, CD34, CD45, CCR2, CXCR4, and collagen-I [6], [7], [9], [11], [13], [16], [20], [22]. We confirmed that human fibrocytes (50–200 µm long spindle-shaped cells with an oval nucleus), cultured for 7 days in SFM, expressed CD13, CD34, CD45RB (the intermediate molecular weight isoform of CD45), CD45RO (the low molecular weight isoform of CD45), CXCR4, and had undetectable levels of CD14 and CD45RA (the high molecular weight isoform of CD45) (Figures 1 and 2).

**Figure 3:**
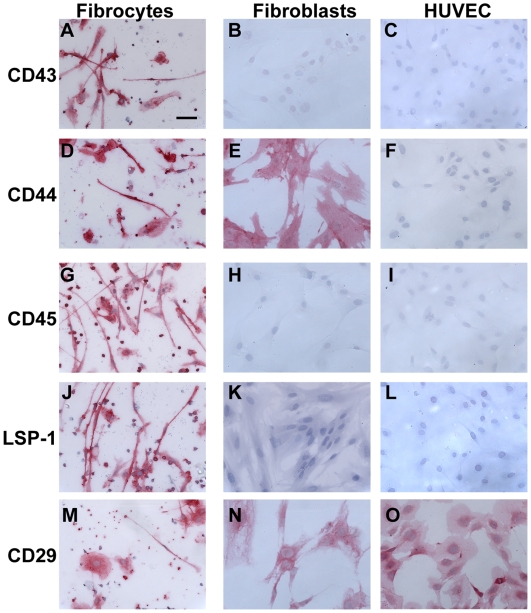
Expression of markers on fibrocytes, fibroblasts, and endothelial cells. PBMC were cultured as described in Figure 1. Normal human dermal fibroblasts and HUVECs were cultured for 2 days in 8-well glass slides. Cells were then air-dried, fixed, and stained with antibodies against A–C) CD43, D–F) CD44, G–I) CD45, J–L) LSP-1, and M–O) CD29. Cells were then counterstained with hematoxylin to identify nuclei. Bar is 50 µm.

As PBMC cultured in SFM for 7 days contain lymphocytes, fibrocytes, and *in vitro* differentiated macrophages, we were able to compare directly the expression of markers on these cell types in the same cultures. As reported previously, we found that lymphocytes (8–10 µm cells, with little or no cytoplasm), *ex vivo* monocytes (10–15 µm cells, with a bilobed nucleus), *in vitro* cultured macrophages (15–20 µm cells, with a large nucleus and pronounced cytoplasm), and fibrocytes, all express CD43, CD44, CD45, LSP-1, CD29 (β1 integrin), and CD18 (β2 integrin) (Figures 1 and 3) [6], [7], [9], [16], [25]. We then performed a screen using commercially available reagents to determine what markers were expressed by fibrocytes, compared to freshly isolated PBMC, *in vitro* cultured macrophages, fibroblasts, and endothelial cells. The data are summarized in Figure 1; key observations made with some of the markers are described below.

### Expression of monocyte-macrophage lineage markers on fibrocytes

**Figure 4:**
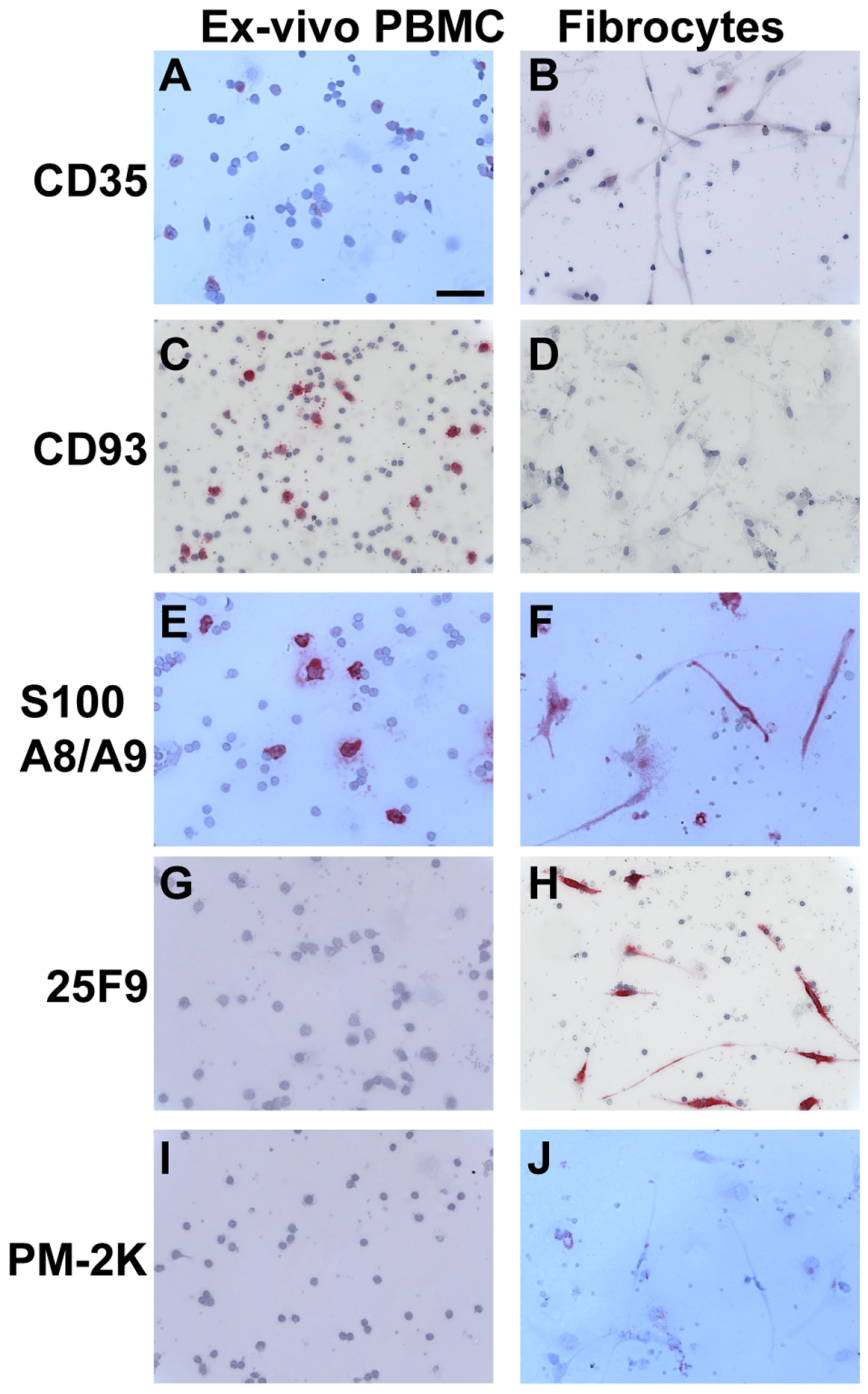
Comparison of markers on PBMC *ex-vivo* and fibrocytes. PBMC were cultured in SFM at 5×10^5^ cells/ml in 8-well glass slides for 1 hour (*ex vivo*) or 7 days. Cells were then air-dried, fixed, and stained with antibodies. A and B) CD35, C and D) CD93, E and F) S100A8/9, G and H) 25F9, and I and J) PM-2K. Cells were then counterstained with hematoxylin to identify nuclei. Bar is 50 µm.

**Figure 5:**
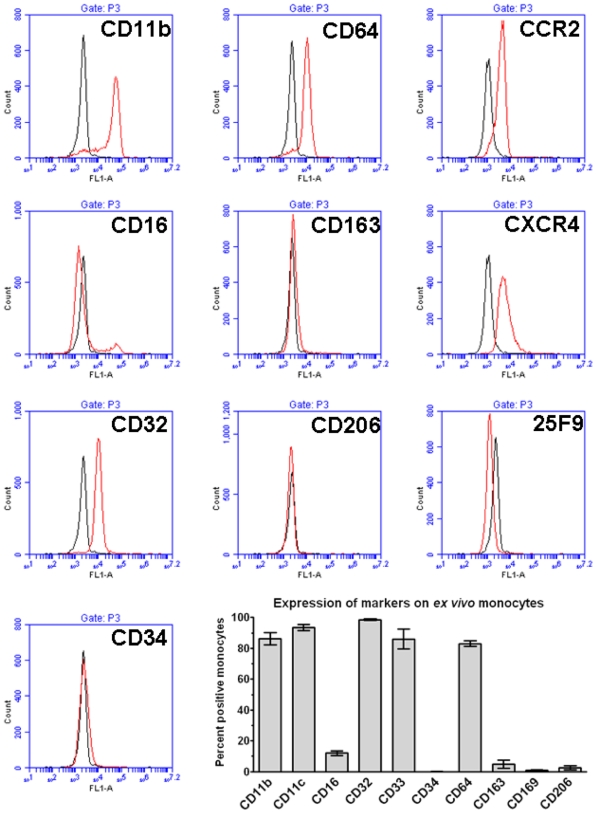
Detection of surface markers on monocytes by flow cytometry. PBMC were stained with mouse monoclonal antibodies and then analyzed by flow cytometry. Monocytes were identified by their forward and side scatter characteristics and the expression of CD14. Histograms show fluorescence intensity of isotype control antibody (black line) compared to the indicated marker (red line) on monocytes. Flow cytometry plots are representative results from six separate donors. Graph shows the percent positive monocytes expressed as the mean±SEM (n = 6 separate donors).

**Figure 6:**
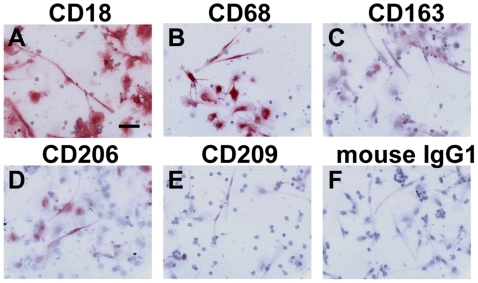
Expression of monocyte/macrophage markers on fibrocytes. PBMC were cultured as described in Figure 1. Cells were then air-dried, fixed, and stained with antibodies against A) CD18, B) CD68, C) CD163, D) CD206, E) CD209, and F) mouse IgG1 control. Cells were then counterstained with hematoxylin to identify nuclei. Bar is 50 µm.

**Figure 7:**
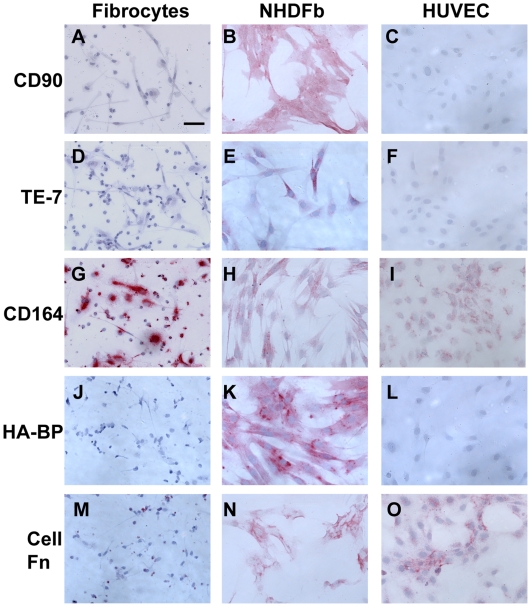
Comparison of fibroblast markers on fibrocytes, fibroblasts, and HUVECs. PBMC were cultured as described in Figure 1. Normal human dermal fibroblasts (NHDFb) and HUVECs were cultured for 2 days in 8-well glass slides. Cells were then air-dried, fixed, and stained A–C) for CD90, D–F) for TE-7, G–I) for CD164, J–L) with HA-BP, and M–O) for cellular fibronectin. Cells were then counterstained with hematoxylin to identify nuclei. Bar is 50 µm.

**Figure 8:**
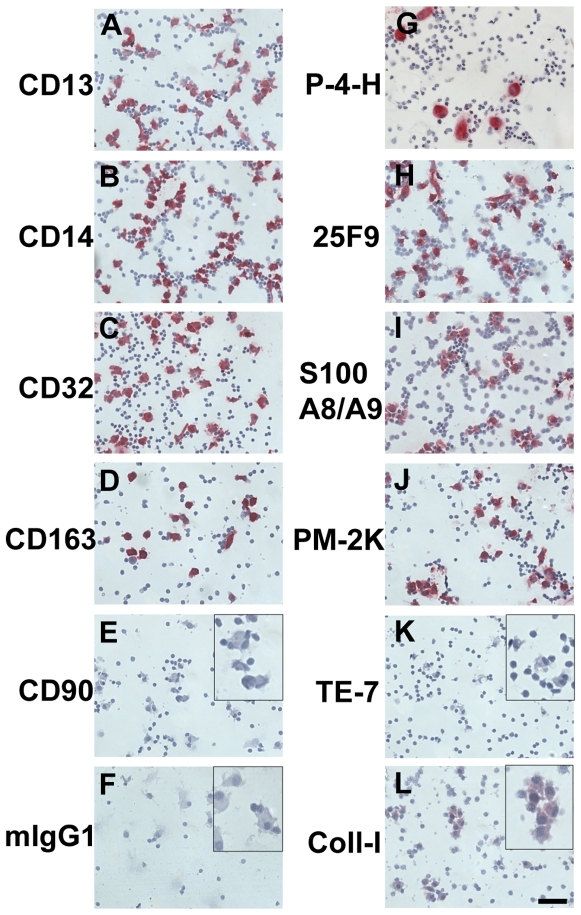
Expression of markers on macrophages cultured in serum and M-CSF. PBMC were cultured in SFM at 5×10^5^ cells/ml in 8-well glass slides for 7 days in the presence of 10% FCS and 10 ng/ml M-CSF. Cells were then air-dried, fixed and stained with 5 µg/ml mouse IgG1 monoclonal antibodies against CD13, CD14, CD32, CD163, CD90, mouse IgG1, proyly-4-hydroxylase, 25F9, S100A8/A9, PM-2K, TE-7, and collagen-I. Cells were then counterstained with hematoxylin to identify nuclei. Inserts indicate staining at higher magnification. Bar is 50 µm.

We first assessed the expression of CD11b, CD11c, CD33, CD35, CD93, calcium binding S100A8/A9 complex (MRP8/14; calprotectin), 25F9, and PM-2K, markers differentially expressed by monocytes and macrophages [64]–[70]. As shown previously, we found that *ex vivo* peripheral blood monocytes, incubated for 60 minutes *in vitro* and identified by their size, expression of CD14, and a bilobed nucleus, expressed CD33, CD35, CD93, and S100A8/A9 (Figures 1 and 4). Macrophages and fibrocytes cultured for 7 days, had low or undetectable expression of CD33, CD35, and CD93, but retained expression of S100A8/A9 (Figures 1 and 4). The 25F9 antigen, which is absent on monocytes but expressed by macrophages, was also present on fibrocytes (Figure 4). PM-2K, a marker expressed only by mature macrophages, was absent on fibrocytes (Figure 4; and see below). The relative surface expression of CD11b, CD11c, CD33, CD35, and 25F9 on peripheral blood monocytes *ex vivo* was confirmed by flow cytometry (Figure 5 and data not shown). Both *in vitro* cultured macrophages and fibrocytes expressed the sialomucins CD68 and CD164, the C-type lectins CD206, CD209, and the scavenger receptor CD163 (Figures 6 and 7). The surface expression of CD163, CD169, CD206, and CD209 was either absent or low on peripheral blood monocytes, as determined by flow cytometry (Figure 5 and data not shown). Macrophages express non-specific esterase (NSE) staining [71]. Both macrophages and fibrocytes were NSE positive, but in macrophages NSE activity was fluoride insensitive whereas in fibrocytes the NSE activity was fluoride sensitive (data not shown). We also cultured PBMC for 7 days in the presence of 10% FCS and 10 ng/ml M-CSF, which are standard conditions for the culture of human macrophages [55]–[58]. We found that macrophages cultured in 10% fetal calf serum and 10 ng/ml M-CSF expressed CD11b, CD11c, CD13, CD14, CD32, CD68, CD163, CD164, CD169, S100A8/A9, 25F9, and PM-2K, but CD33 and CD35 were undetectable (Figure 8 and data not shown). There was no discernable difference in the expression of CD13, CD68, CD163, S100A8/A9, and 25F9, between macrophages cultured in SFM compared to FCS and M-CSF (Figures 2, 4, 6 and 8). However, we did observe that CD14 and PM-2K were expressed on macrophages cultured in the presence of serum and M-CSF. These results suggest that CD33, CD35, CD93, and 25F9 expression can be used to distinguish monocytes from macrophages and fibrocytes, and that fluoride-sensitive NSE activity and PM-2K antigen expression may discriminate fibrocytes from tissue macrophages.

### Expression of FcγR receptors

**Figure 9:**
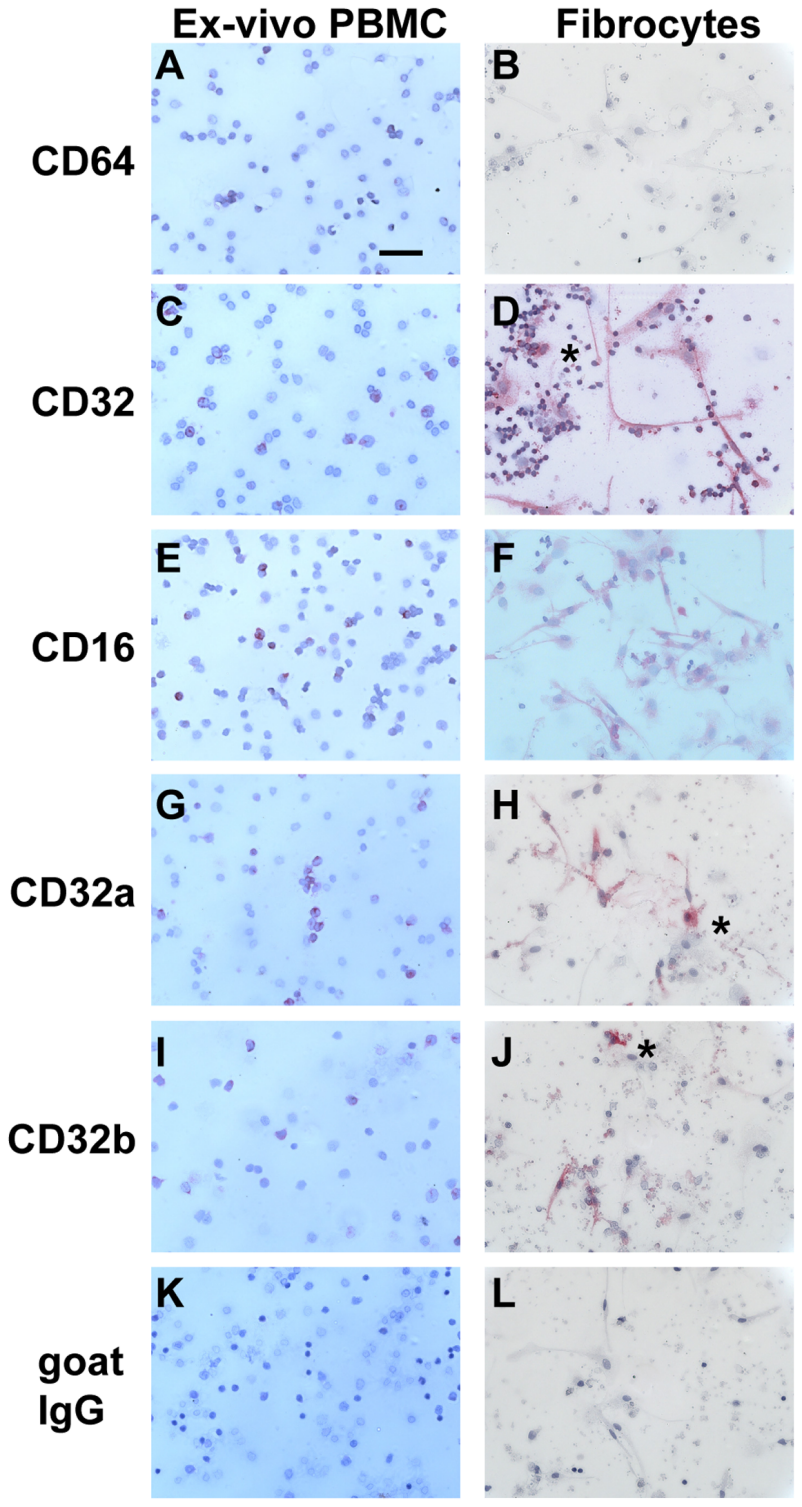
Comparison of Fc receptors on PBMC *ex-vivo* and fibrocytes. PBMC were cultured in SFM at 5×10^5^ cells/ml in 8-well glass slides for 1 hour (*ex vivo*) or 7 days. Cells were then air-dried, fixed, and stained with antibodies against A and B) CD64, C and D) CD32, E and F) CD16, G and H) CD32a, I and J) CD32b, or K and L) goat IgG control antibodies. Cells were then counterstained with hematoxylin to identify nuclei. Asterisks are to the right of macrophages. Bar is 50 µm.

We previously found that the inhibition of fibrocyte differentiation by SAP may act through FcγR ligation [17], [72]. Therefore, we examined the expression of FcγR on fibrocytes and macrophages, compared to *ex-vivo* peripheral blood monocytes. As described previously, monocytes express CD64 (FcγRI) and CD32 (FcγRII), with a subpopulation of monocytes also positive for CD16 (FcγRIII) (Figure 9) [70], [73]. The expression of CD16, CD32, and CD64 on peripheral blood monocytes was confirmed by flow cytometry (Figure 5). We found that after 7 days in SFM culture, macrophages expressed CD16 (FcγRIII) and CD32 (FcγRII), but CD64 (FcγRI) expression was undetectable (Figure 9). Macrophages in populations of PBMC cultured in the presence of FCS and M-CSF expressed predominantly CD32 (Figure 8). Fibrocytes expressed both CD32a and CD32b isoforms, but CD16 expression was low and CD64 expression was undetectable (Figure 9). Both fibroblasts and endothelial cells had no detectable FcγR expression (Figure 1 and data not shown).

### Expression of stem cell markers

Compared to peripheral blood monocytes, fibrocytes express CD34 (Figures 1 and 5) [6], [16]–[18], [74]. CD34 is not only a classical stem cell marker, but a member of the sialomucin family of receptors, that also includes CD43, CD68, and CD164, which fibrocytes also express (Figures 3, 6, and 7) [33], [75]. CD133, CD150, and STRO-1 are also markers associated with hematopoietic and mesenchymal stem cells, but these markers did not appear to be expressed by fibrocytes (Figure 1 and data not shown). The high molecular weight isoform of CD45, CD45-B220, which is the heavily N- and O-glycosylated form of CD45, and has been reported to be a marker of certain stem cell populations [76]. We did not detect the expression of CD45-B220 on monocytes, macrophages, or fibrocytes (Figure 1).

### Expression of stromal cell markers

We then examined markers associated with stromal cells. Fibroblasts and endothelial cells had no detectable staining for CD43, CD45, and LSP-1, although fibroblasts stained for CD29, and CD44, and endothelial cells were CD29, CD93 and vWF positive (Figures 1 and 3, and data not shown). Unlike MRC-5 fetal fibroblasts and normal human dermal fibroblasts (NHDF), fibrocytes and macrophages had no detectable staining for CD90, CD248 (endosialin), FAP, TE-7, cellular fibronectin, or hyaluronan (HA-BP) (Figures 1, 7, and 8). We were also able to confirm that normal human dermal fibroblasts and human fetal fibroblasts (MRC-5) also express CD68 (originally described as a monocyte/macrophage marker) and CD164 (Figures 1 and 7), as described previously [51]–[54]. Fibroblasts also expressed tenascin, and endothelial cells expressed cytokeratin, but these markers were undetectable on fibrocytes (Figure 1 and data not shown). These data indicate that there are at least six commercial reagents (cellular fibronectin, CD90, TE-7, CD248, HA-BP, and FAP) that can be used to discriminate between fibrocytes and fibroblasts.

### Expression of collagen matrix markers

**Figure 10:**
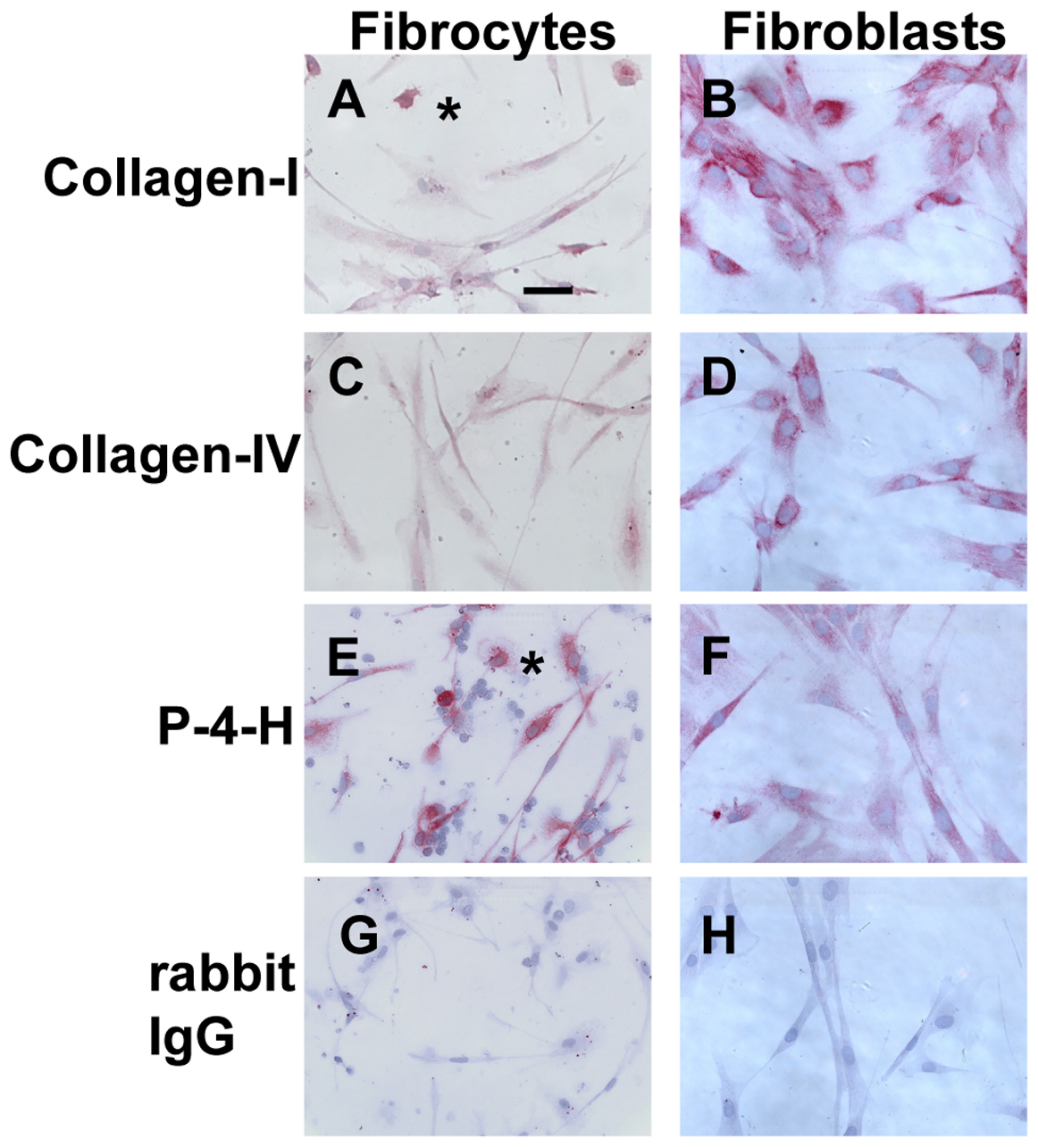
Comparison of collagen expression on fibrocytes and fibroblasts. PBMC were cultured as described in Figure 1. Normal human dermal fibroblasts were cultured for 2 days in 8-well glass slides. Cells were then air-dried, fixed, and stained with antibodies. A and B) collagen-I, C and D) collagen IV, E and F) proyly-4-hydroxylase, G and H) rabbit IgG control antibody. Cells were then counterstained with hematoxylin to identify nuclei. Asterisks are to the right of macrophages. Bar is 50 µm.

Fibrocytes showed positive staining for collagen-I, collagen-IV, pro-collagen-I, and prolyl-4-hydroxylase (a key enzyme in collagen synthesis), but the collagen staining was much weaker than that found on fibroblasts (Figure 1 and 10). We also detected weak collagen staining on macrophages cultured in either SFM or with serum and M-CSF, compared to cells incubated with irrelevant IgG (Figures 8 and 10). These data suggest that either the macrophages were able to produce low levels of collagen, or that the macrophages were able to ingest collagen from other sources in the culture. The source of collagen is unlikely to be from the serum-free medium, and is more likely to be from either collagen bound to the cells before blood collection, or collagen-secreting cells in the cultures. Antibodies against prolyl-4-hydroxylase or pro-collagen I only label cells actively synthesizing collagen. There were low levels of staining on macrophages, cultured in either SFM or with serum and M-CSF, with both prolyl-4-hydroxylase and collagen-I antibodies (Figures 1, 8 and 10), indicating that macrophages may produce collagen, as described previously [34], [38]. These data suggest that the use of collagen staining to discriminate CD45/CXCR4 or CD34/CD45 positive macrophages from CD45/CXCR4/collagen positive or CD34/CD45/collagen positive fibrocytes may be insufficient to discriminate macrophages from fibrocytes.

### Expression of lectin and carbohydrate residues

**Figure 11:**
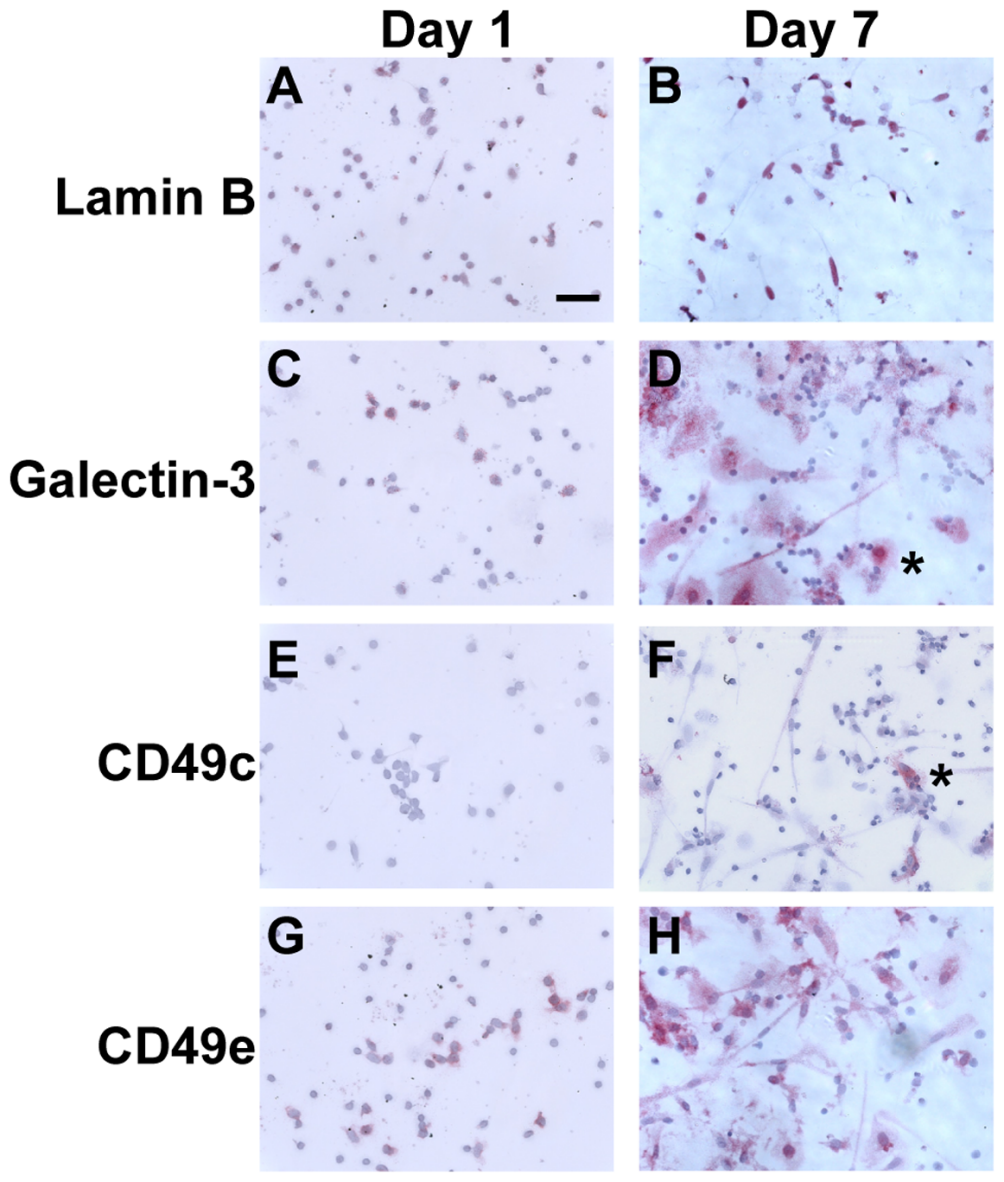
Expression of markers on fibrocytes cultured for 21 days. PBMC were cultured in SFM at 5×10^5^ cells/ml in 8-well glass slides for 21 days. Cells were then air-dried, fixed, and stained with antibodies. A) PM-2K, B) CD18, C) CD34, D) CD68, E) CD163, F) CD164, G) CD169, H) CD49c, and I) CD49e. Cells were then counterstained with hematoxylin to identify nuclei. Bar is 50 µm. Graph shows the percent positive CD49c (α3 integrin) expression over 21 days in culture. At least 100 elongated cells with oval nuclei were examined from at least 10 randomly selected fields, and the percentage of elongated cells that were positively stained by antibodies against the indicated markers is expressed as the mean±SEM (n = 5 separate donors). *** indicates p<0.001, as determined by ANOVA.

As the expression of carbohydrate residues on glycoproteins change as cells differentiate [77], we assessed whether fibrocytes expressed different carbohydrates compared to monocytes and macrophages. Monocytes stained with the lectins wheat germ agglutinin (WGA) and peanut agglutinin (PNA), but not soybean agglutinin (SBA), whereas macrophages and fibrocytes retained the expression of WGA, but did not stain with PNA or SBA (data not shown). Mac2/Galectin-3 is a lectin binding receptor expressed by macrophages [78]. Both macrophages and fibrocytes expressed Mac2/Galectin 3 (Figure 1 and 11).

### Expression of markers on long-term cultured fibrocytes

**Figure 12:**
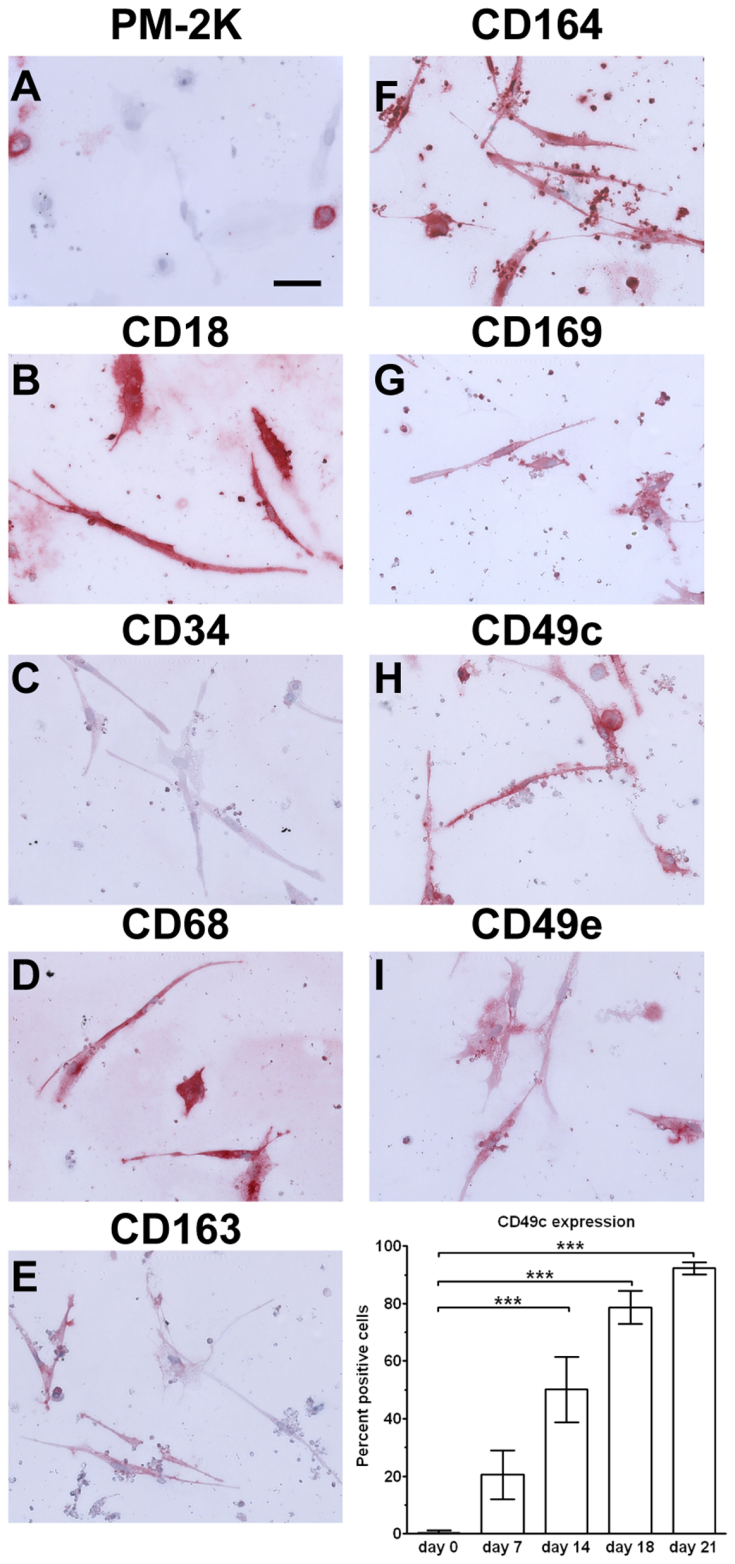
Comparison of markers on PBMC cultured for 1 day and day 7 fibrocytes. PBMC were cultured in SFM at 5×10^5^ cells/ml in 8-well glass slides for 1 or 7 days. Cells were then air-dried, fixed, and stained with antibodies. A and B) Lamin B, C and D) galectin-3, E and F) CD49c, and G and H) CD49e. Cells were then counterstained with hematoxylin to identify nuclei. Asterisks indicate macrophages. Bar is 50 µm.

We next addressed whether markers expressed by fibrocytes changed when PBMC were cultured *in vitro* for up to three weeks. We found that long-term cultured fibrocytes also expressed many of the markers expressed by fibrocytes cultured for 1 week, such as CD18, CD43, CD44, CD45RO, CD49e, CD68, CD163, CD164, CD169, LSP-1, and CXCR4 (Figure 12 and data not shown). We observed a reduced expression of CD34, as described previously (Figure 12) [20]. We also observed that after 3 weeks in culture, compared to macrophages fibrocytes had undetectable expression of CD14 and PM-2K (Figures 2, 4, 8, 12, and data not shown). Using a screen of integrin receptor expression, we found that compared to the expression of CD49e (α5 integrin) on fibrocytes and macrophages, the number of CD49c (α3 integrin) positive fibrocytes increased as cells were cultured for up to three weeks (Figure 11 and 12). These data suggest that PM-2K may be a useful marker to distinguish fibrocytes from macrophages, and that CD49c could be a useful marker to distinguish mature fibrocytes from younger fibrocytes.

### Expression of markers on “early” fibrocytes

We next assessed whether we could identify markers that could discriminate cells that had the potential to differentiate into fibrocytes. We screened PBMC at time points before fibrocytes appeared in the cultures. As described previously, compared to *ex vivo* monocytes (Figures 4 and 5), monocytes cultured *in vitro* for 24 hours had low to undetectable levels of CD14 and CD93 (data not shown) [69], [79]. We did detect the expression of 25F9 antigen on PBMC cultured for 2 days, as described previously (Figure 1) [67]. We were also unable to detect collagen, a marker of mature fibrocytes, by early fibrocyte-like cells (data not shown). Finally, we assessed whether PU.1 and myeloid antigen, two transcription factors expressed in monocyte precursors [80], [81], were expressed by cells during the early stages of monocyte to fibrocyte differentiation. In PBMC cultured for up to seven days in serum free medium, we did not detect PU.1 or myeloid antigen (Figure 1). The most characteristic changes associated with early fibrocyte differentiation were nuclear shape change from a kidney bean shape to a small oval nucleus, and extensive cytoskeletal rearrangements as assessed by tubulin and lamin B staining (Figure 11 and data not shown). These data suggest that currently the earliest detectable changes in monocytes differentiating into fibrocytes are morphological changes, rather than specific changes in receptor expression.

### Expression of markers on fibrocytes exposed to cytokines

**Figure 13:**
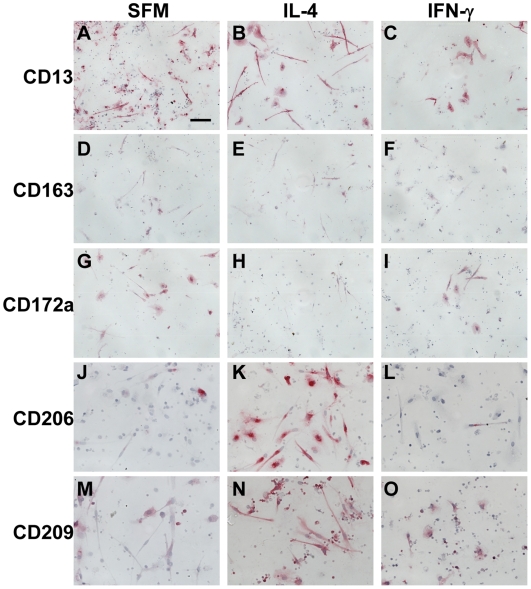
Effect of cytokines on markers expressed by fibrocytes. PBMC were cultured in 8-well glass slides in SFM for 7 days, in the presence or absence of IL-4, or IFN-γ. Cells were then air-dried, fixed, and stained with antibodies. A–C) CD13, D–F) CD163, G–I) CD172a, J–L) CD206, and M–O) CD209. Cells were counterstained with hematoxylin to identify nuclei. Bar is 50 µm.

Both *in vitro* and *in vivo* activated macrophages express different markers when exposed to cytokines, immune complexes, or bacterial products [82]–[84]. Therefore, we assessed whether fibrocytes also differentially expressed markers in the presence or absence of IL-4, IL-12, IL-13, and IFN-γ, molecules known to promote or inhibit fibrocyte differentiation [18]. PBMC cultured in SFM containing IL-4 (or IL-13, data not shown) had increased numbers of fibrocytes, as described previously [18], and these fibrocytes stained more intensely for CD206 and CD209, and had reduced expression of CD172a, compared to fibrocytes cultured in SFM alone (Figure 13). However, for PBMC cultured in SFM containing IFN-γ (or IL-12, data not shown), the few fibrocytes observed retained CD172a expression, but had variable expression of CD206 and CD209 (Figure 13). The presence or absence of IL-4, IL-12, IL-13, or IFN-γ had no apparent effect on the expression of other markers associated with fibrocytes and macrophages, such as CD13, CD68, CD163, S100A8/A9, or 25F9 (Figure 13, and data not shown). These data suggest that as previously described for macrophages, the markers CD172a, CD206, and CD209 can be used to determine whether fibrocytes have differentiated in an environment containing pro-fibrotic Th-2-like cytokines (IL-4 or IL-13) compared to Th-1-like pro-inflammatory cytokines (IL-12 or IFN-γ) [84], [85].

### Expression of markers on fibrocytes exposed to SAP

**Figure 14:**
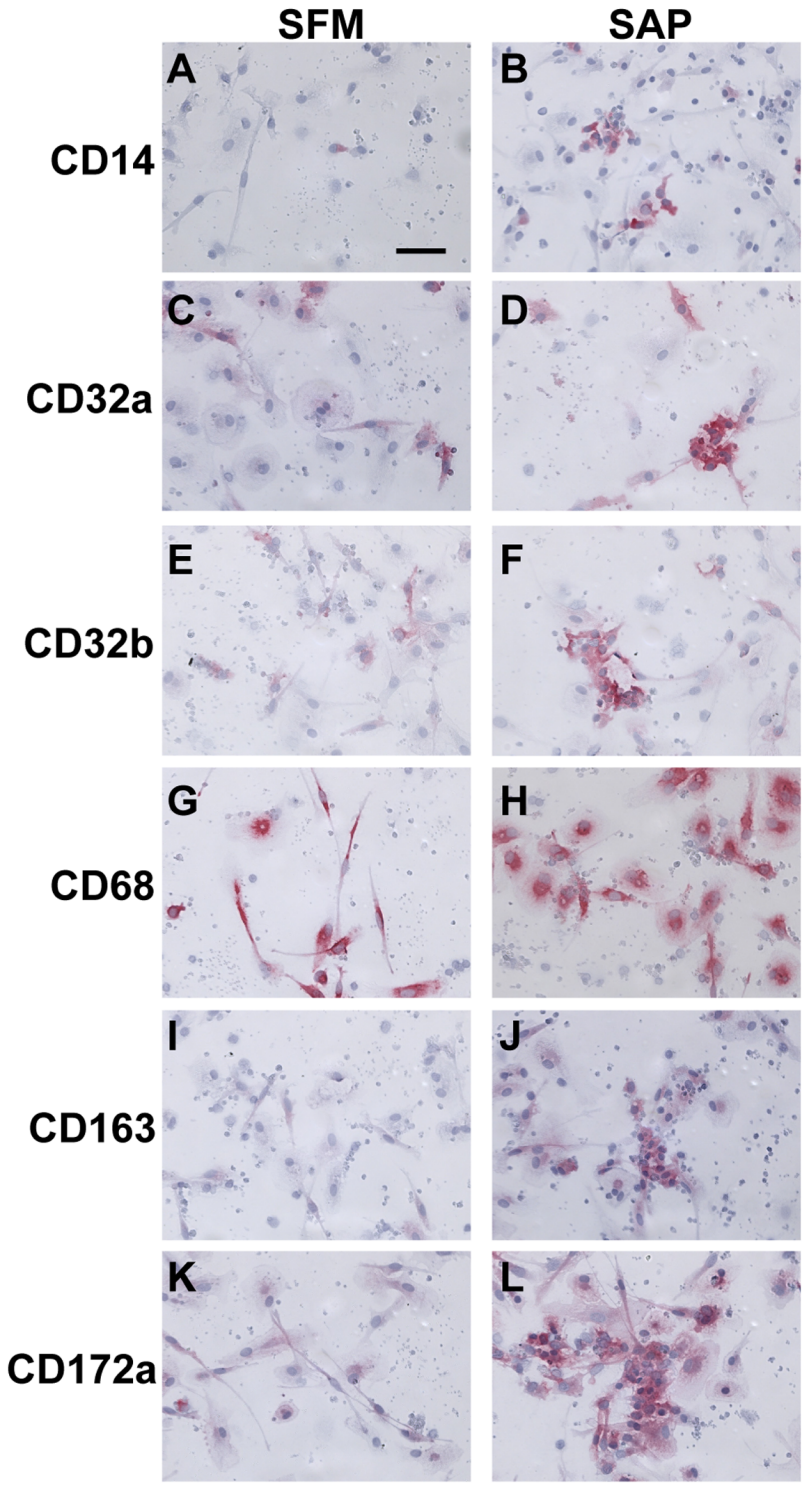
Effect of SAP on markers expressed by macrophages. PBMC were cultured in 8-well glass slides in SFM for 7 days, in the presence or absence of SAP. Cells were then air-dried, fixed, and stained with antibodies. A–B) CD14, C–D) CD32a, E–F) CD32b, G–H) CD68, I–J) CD163, and K–L) CD172a. Cells were counterstained with hematoxylin to identify nuclei. Bar is 50 µm.

We have previously shown that SAP inhibits fibrocyte differentiation *in vitro* and *in vivo*, and that this process appears to be dependent on the FcRγ chain, a common membrane-signaling component of CD64, CD32a, and CD16 [13], [16], [17], [72]. We therefore assessed whether PBMC differentially expressed markers cultured in the presence or absence of SAP. We first assessed whether the presence of SAP affected the loss of CD93 and gain of 25F9 expression on PBMC, two markers that change rapidly when cultured *in vitro* (Figure 4 and data not shown). We found that in the presence or absence of SAP after 2 days, CD93 expression was undetectable, and that 25F9 expression was detectable in cells (data not shown). We than assessed whether SAP altered the expression of markers on PBMC cultured for 7 days. PBMC cultured in SFM containing SAP had reduced numbers of fibrocytes, as described previously [16], [17]. PBMC cultured in the presence of SAP had increased expression of CD14, CD32a, CD32b, CD163, and CD172a on the macrophages, compared to cells cultured in SFM alone, whereas markers such as CD45, CD68, CD164, and PM-2K were unaltered (Figure 14 and data not shown). These data suggest that the markers CD14, CD32, CD163, and CD172a, might be used to determine whether macrophages have differentiated in an environment containing SAP.

### Identification of fibrocytes in pathological samples

**Figure 15:**
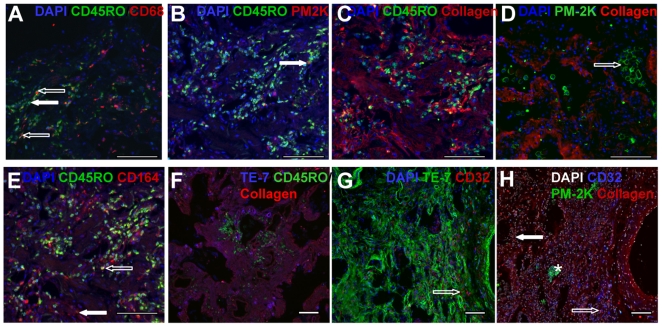
Identification of fibrocytes in pathological samples. “HOPE-fixed” human lung tissue sections were stained with antibodies against CD32, CD45RO, CD68, CD164, collagen-I, PM-2K, and TE-7. Sections were stained for A) CD45RO (green), CD68 (red) and DAPI (blue). An example of a CD45RO positive lymphocyte is indicated with a solid arrow. Dual labeled CD45RO and CD68 macrophages and fibrocytes are indicated by open arrows. B) CD45RO (green), PM-2K (red) and DAPI (blue). An example of a dual labeled CD45RO and PM-2K tissue macrophage is indicated with a solid arrow. C) CD45RO (green), collagen-I (red), and DAPI (blue). Dual labeled CD45RO and collagen-I positive cells are yellow. D) PM-2K (green), collagen-I (red), and DAPI (blue). A cluster of PM-2K positive alveolar macrophages is indicated with an open arrow. E) CD45RO (green), CD164 (red), and DAPI (blue). An area of CD164 positive fibroblasts is indicated with a solid arrow. A dual labeled CD45RO and CD164 positive fibrocyte is indicated by an open arrow. F) CD45RO (green), collagen-I (red), and TE-7 (blue). Dual positive TE-7/collagen-I fibroblasts are magenta. G) TE-7 (green), CD32 (red), and DAPI (blue). An example of CD32 positive fibrocytes is indicated with an open arrow. H) Nuclei (white), PM-2K (green), collagen (red), and CD32 (blue). A cluster of PM-2K positive alveolar macrophages are indicated with an asterisk. An area of CD32 and collagen positive/PM-2K negative fibrocytes is indicated with an open arrow. An area of collagen positive/CD32 negative fibroblasts is indicated with a solid arrow. Cells were counterstained with DAPI to identify nuclei. Bars are 100 µm.

Finally, we assessed whether the markers we identified as specific for monocytes, macrophages, fibroblasts, and fibrocytes using *in vitro* cultured cells were valid for human pathological samples. We stained sections of human lung tissue from patients with chronic obstructive pulmonary disease (COPD) and interstitial lung disease (ILD) with antibodies against collagen-I, CD32, CD45RO, CD68, CD93, CD164, LSP-1, PM-2K, and TE-7 (Figure 15 and data not shown). Staining lung tissue from COPD patients with CD45RO and CD68 antibodies showed the presence of both small CD45RO positive/CD68 negative cells, which appear to be lymphocytes, and CD45RO/CD68 dual positive macrophages and fibrocytes (Figure 15A). We also observed CD45RO negative/CD68 positive fibroblasts (Figure 15A). Using CD45RO and PM-2K staining we were also able to discriminate the small number of CD45RO/PM-2K dual positive tissue macrophages from PM-2K negative CD45RO positive lymphocytes and fibrocytes (Figure 15B). Collagen-I expression was present throughout the lung, with distinct areas containing CD45RO and collagen-I dual positive cells (Figures 15C). We also noted that the majority of PM-2K positive cells were localized in the alveolar spaces (Figure 15D), suggesting that they are alveolar macrophages rather than interstitial macrophages, and that PM-2K positive cells did not appear to express collagen (Figure 15D). We also identified fibroblasts as CD45RO negative/CD164 positive cells, and fibrocytes as CD45RO/CD164 positive cells (Figure 15E). Sections stained with CD45RO, TE-7 and collagen showed large areas of TE-7/collagen dual positive fibroblasts, and the absence of TE-7 staining on CD45RO positive cells (Figure 15F). Sections stained with CD32 and TE-7 showed a network of TE-7 positive cells (Figure 15G), whereas CD32 was restricted to alveolar and interstitial macrophages. We did not detect any cells that were stained for CD32 and TE-7, suggesting that these two antibodies label distinct cell types, and that CD32 is an additional marker to discriminate fibrocytes in fibrotic lesions (Figure 15G). Finally, using DAPI, CD32, PM-2K, and collagen we were able to use four color fluorescence to discriminate macrophages (PM-2K/CD32 positive), fibroblasts (collagen positive but CD32 and PM-2K negative), and fibrocytes (CD32/collagen dual positive but PM-2K negative) (Figure 15H). These data suggest that the expression of PM-2K is absent on fibrocytes, and that CD164 and TE-7 expression can be used to identify fibroblasts. Finally, fibrocytes can be identified by using a combination of antibodies to PM-2K, CD32, CD45RO, and collagen.

## Discussion

In this report, we stained human monocytes, macrophages, fibrocytes, and fibroblasts with a large panel of antibodies to find markers that can distinguish these cells. We found that we can exploit the expression of PM-2K on macrophages and the lack of PM-2K expression on fibrocytes to distinguish fibrocytes from macrophages in human *in vitro* cultured cells and human lung tissue. We were also able to discriminate collagen positive fibrocytes from collagen positive fibroblasts using the expression of cellular fibronectin, HA-BP or TE-7 on fibroblasts but not fibrocytes. Finally, we found that CD49c (α3 integrin) appears to be a marker of fibrocytes that appear after an extended period in culture.

The PM-2K antibody binds to a 150 kD cytoplasmic protein that appears to be specific for tissue macrophages, but the gene has yet to be identified [68]. PM-2K has previously been shown to stain mature tissue macrophages in humans and non-rodent mammals [68], [86], [87]. Due to the differential expression of PM-2K on macrophages and fibrocytes, we would suggest that PM-2K expression should be used as a criterion to distinguish these two cell types, along with morphology and fluoride-sensitive esterase staining.

Several groups have shown that fibrocytes have a progressive loss of hematopoietic markers as they differentiate from monocytes [9], [20], [24]. Although we did not detect a loss of CD45RO on fibrocytes, we did detect the loss of CD34. However, we observed that as monocytes differentiate into fibrocytes, there is an increased expression of Mac-2/galectin 3, and long-term cultured fibrocytes express CD49c. These markers may by useful to discriminate fibrocytes (CD34/CD45RO positive but CD49c negative spindle-shaped cells with an oval nucleus) from late differentiating fibrocytes (CD45RO/CD49c positive but CD34 negative spindle-shaped cells with an oval nucleus). Alternatively, the expression of CD49c may identify a population of fibrocytes that differentiate from monocytes only after an extended period in culture.

It is difficult to discriminate fibrocytes with low levels of the hematopoietic markers CD34 and CD45 from mesenchymal fibroblasts in tissue sections. We observed that, as reported previously, fibroblasts express CD68, which precludes it as a marker to discriminate fibroblasts from macrophages and fibrocytes [51]–[54]. However, we found that several fibroblast markers such as FAP, TE-7, cellular fibronectin, or hyaluronan, are undetectable on fibrocytes. Therefore, we would recommend the use of one these markers as a criterion to distinguish fibrocytes from fibroblast.

We have also been able to show that the loss of CD33, CD35, CD93, and PNA expression on monocytes and the gain of 25F9 expression on macrophages can be used to differentiate these two cell types. The loss of CD33, CD35, CD93, PNA, and CD14, and the gain of 25F9, CD34 and CD49c will distinguish monocytes from fibrocytes. Finally, the expression of CD11a, CD11b, CD11c, CD18, CD43, CD45, and LSP-1 on monocytes, macrophages, and fibrocytes, and the expression of CD90, hyaluronan, and fibronectin on fibroblasts distinguish these two sets of cells.

For our analysis of fibrocyte markers, we used commercially available reagents to remove the issue of limited availability of antibodies from non-commercial sources. This has the added advantage that differences in the expression patterns or numbers of cells between laboratories is less likely to be due to variations in antibody preparation, and more likely to represent variation due to tissue processing or an actual variation in the biological process.

These studies indicate that the discrimination of monocytes, macrophages, fibrocytes, and fibroblasts is possible. In particular, the accurate discrimination of macrophages, fibrocytes, and fibroblasts will be beneficial in quantifying these cells in fibrotic lesions to determine if an increased number of one or more of these cell types is associated with a fibrotic disease.
